# Robot-assisted pyeloplasty and laparoscopic pyeloplasty in children: A comparison of single-port-plus-one and multiport surgery

**DOI:** 10.3389/fped.2022.957790

**Published:** 2022-10-21

**Authors:** Jianglong Chen, Huihuang Xu, Shan Lin, Shaohua He, Kunbin Tang, Zhixiang Xiao, Di Xu

**Affiliations:** ^1^ Shengli Clinical Medical College, Fujian Medical University, Fuzhou, China; ^2^ Department of Pediatric Surgery, Fujian Provincial Hospital, Fuzhou, China

**Keywords:** UPJO, pyeloplasty, robotic, single-port, pediatric

## Abstract

**Objective:**

This study aimed to compare the effects of various trocar placements in robot-assisted and laparoscopic pyeloplasty involving children diagnosed with obstruction of the ureteropelvic junction (OUPJ).

**Methods:**

We retrospectively collected the data on 74 patients under 14 years of age who had been diagnosed with OUPJ; these patients underwent either robot-assisted or laparoscopic pyeloplasty in our hospital between January 2015 and November 2021. There were four groups, as follows:

• Laparoscopic multiport pyeloplasty (LMPY),
• Laparoscopic single-port pyeloplasty (LSPY),
• Robotic-assisted multiport pyeloplasty (RMPY),
• Robotic-assisted single-port-plus-one pyeloplasty (RSPY).

Patients' characteristics as well as their perioperative and follow-up data were collected and evaluated.

**Results:**

There was no significant difference in the data regarding patients' characteristics. These data included the grade of hydronephrosis according to the Society of Fetal Urology (SFU grade), anterior and posterior diameter of the renal pelvis and ureter (APDRPU), and the differential degree of renal function (DRF) at following time points: preoperative, postoperative, and comparison of preoperative and postoperative. There was no difference among these groups. During surgery, the time of trocar placement, urethroplasty time, and total operative time in the robotic groups (RMPY and RSPY) were longer than those in the laparoscopic groups (LMPY and LSPY). However, the ratio of the urethroplasty time and full operative time (UT/WT) in the robotic groups (RMPY and RSPY) was lower than that in the laparoscopic groups (LMPY and LSPY) (*P* = 0.0075). Also, the volume of blood loss was lower in the robotic groups (RMPY and RSPY) than that in the laparoscopic groups (LMPY and LSPY), although there was no statistical difference (*P* = 0.11). There were, however, significant differences in hospitalization days (*P* < 0.0001) and parents' cosmetic satisfaction scores (*P* < 0.001). There were no differences in fasting time, the length of time that a ureteral catheter remained in place, or the number of postoperative complications.

**Conclusion:**

Our study shows that both robotic multiple-port and single-port-plus-one approaches are comparable, with laparoscopic multiple-port and single-port approaches equally effective in resolving OUPJ in children. Robotic and single-port-plus-one approaches may be associated with some advantages in hospitalization time and cosmetic outcomes; therefore, these approaches may be useful in urologic surgery that requires precise suturing, especially in pediatric patients.

## Introduction

Pyeloplasty can be performed by an open surgical, laparoscopic, or robot-assisted approach in children diagnosed with obstruction of the ureteropelvic junction (OUPJ). The outcomes of pyeloplasty performed by the laparoscopic and robot-assisted approaches are comparable to those achieved by open surgery (1). Robotic surgery is a form of minimally invasive surgery (MIS), the benefits of which are increasingly being recognized, especially for children. Previous research has demonstrated the benefits of MIS, including better cosmetic outcomes, minimal operative trauma, less postoperative pain, less need for postoperative opioid use, and a shorter length of hospitalization (2, 3). To achieve the goal of MIS, a single port is used, thus reducing the size and number of incisions in both laparoscopic and robotic surgery. However, a single port can lead to the collision of robotic arms and a more complex operative procedure; it also involves a steep learning curve for the surgeon (4, 5). A single-port-plus-one approach, which avoids these shortcomings to some extent, is used in many cases (6, 7).

This study presents the single-port-plus-one trocar placement protocol in robot-assisted pyeloplasty in children and reports the comparative perioperative and follow-up outcomes of the various approaches.

## Materials and methods

### Ethical review and informed consent

This study was approved by the Institutional Review Board of Fujian Provincial Hospital. After the novel robotic system had been explained to them, all patients signed an informed consent form.

### Patients and design

A total of 71 patients were diagnosed with unilateral OUPJ and 3 with bilateral OUPJ. All were under 14 years of age and received either laparoscopic or robot-assisted pyeloplasty at our institution between January 2015 and November 2021, and all procedures were performed by the same surgeon. Indications of OUPJ for surgical treatment included an SFU grade of III or higher, deteriorating renal function, or repetitive urinary tract infections. In terms of surgical approach, the patients were divided into four groups as follows:

• Laparoscopic multiport pyeloplasty (LMPY),
• Laparoscopic single-port pyeloplasty (LSPY),
• Robot-assisted multiport pyeloplasty (RMPY),
• Robot-assisted single-port-plus-one pyeloplasty (RSPY).

The choice of surgical approach was based mainly on the patient's age and weight as well as on the economic status of the parents.

### Surgical techniques

The da Vinci Xi Surgical Robot (Da Vinci, Mountain View, CA, USA) was used in both robot-assisted multiport pyeloplasty and robot-assisted single-port-plus-one pyeloplasty. Multiport and single-port approaches were used in laparoscopic pyeloplasty. A single port (Sunride, Changzhou, Jiangsu Province, China) was used in robot-assisted single-port-plus-one pyeloplasty and laparoscopic single-port pyeloplasty. For RSPY, the observational port, one of the operative arms of the robot and assistant instrument, was placed through the single-port aisles and another outside of the single port as the plus-one trocar. Laparoscopic multiport pyeloplasty and robot-assisted multiport pyeloplasty were performed through three and four intraperitoneal port sites, respectively.

**Figure 1:**
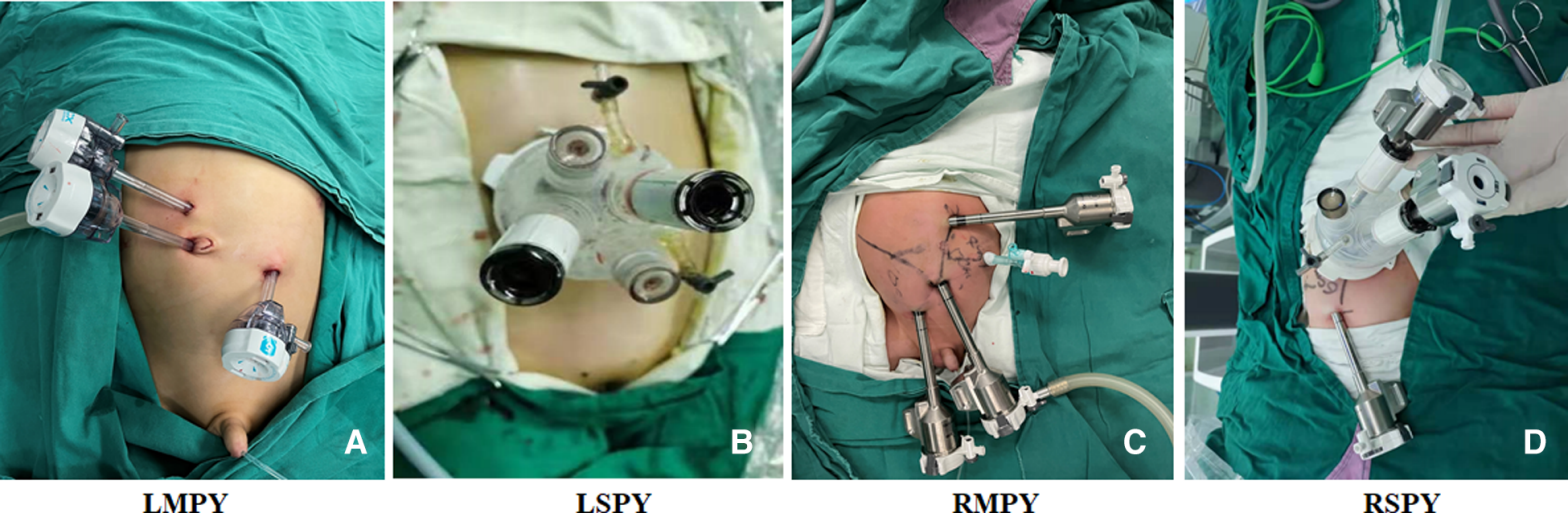
The trocar's location in each group. (**A**) LMPY, laparoscopic multiport pyeloplasty. (**B**) LSPY, laparoscopic single-port pyeloplasty. (**C**) RMPY, robot-assisted multiport pyeloplasty. (**D**) RSPY, robot-assisted single-port-plus-one pyeloplasty.

For RSPY, a 25- to 30-mm umbilical incision was made to insert the trocar and establish the peritoneal cavity. After the artificial pneumoperitoneum was established, another 8-mm robotic trocar was inserted under the direct vision of the camera, and the 8-mm trocar was placed at the left or right abdomen, depending on the surgical site. The assistant's laparoscopic instrument was placed through the aisle of the single port. For RMPY, three robotic 8-mm trocars were placed in (1) the upper abdomen, (2) beside the umbilicus, and (3) the lower abdomen; the assistant's laparoscopic instrument was placed through another 5-mm trocar at the left or right abdomen, depending on the surgical site. For LSPY, a 25- to 30-mm umbilical incision was made to insert a single port and establish the peritoneal cavity, and the operator's two main laparoscopic instruments, the camera, and the assistant's laparoscopic instrument were placed through the single port. For LMPY, four 5-mm trocars were placed at (1) the upper abdomen, (2) beside the umbilicus, and (3) the lower abdomen, while (4) the assistant's trocar was placed at the left or right abdomen, depending on the surgical site, as shown in Figure 1.

**Figure 2:**
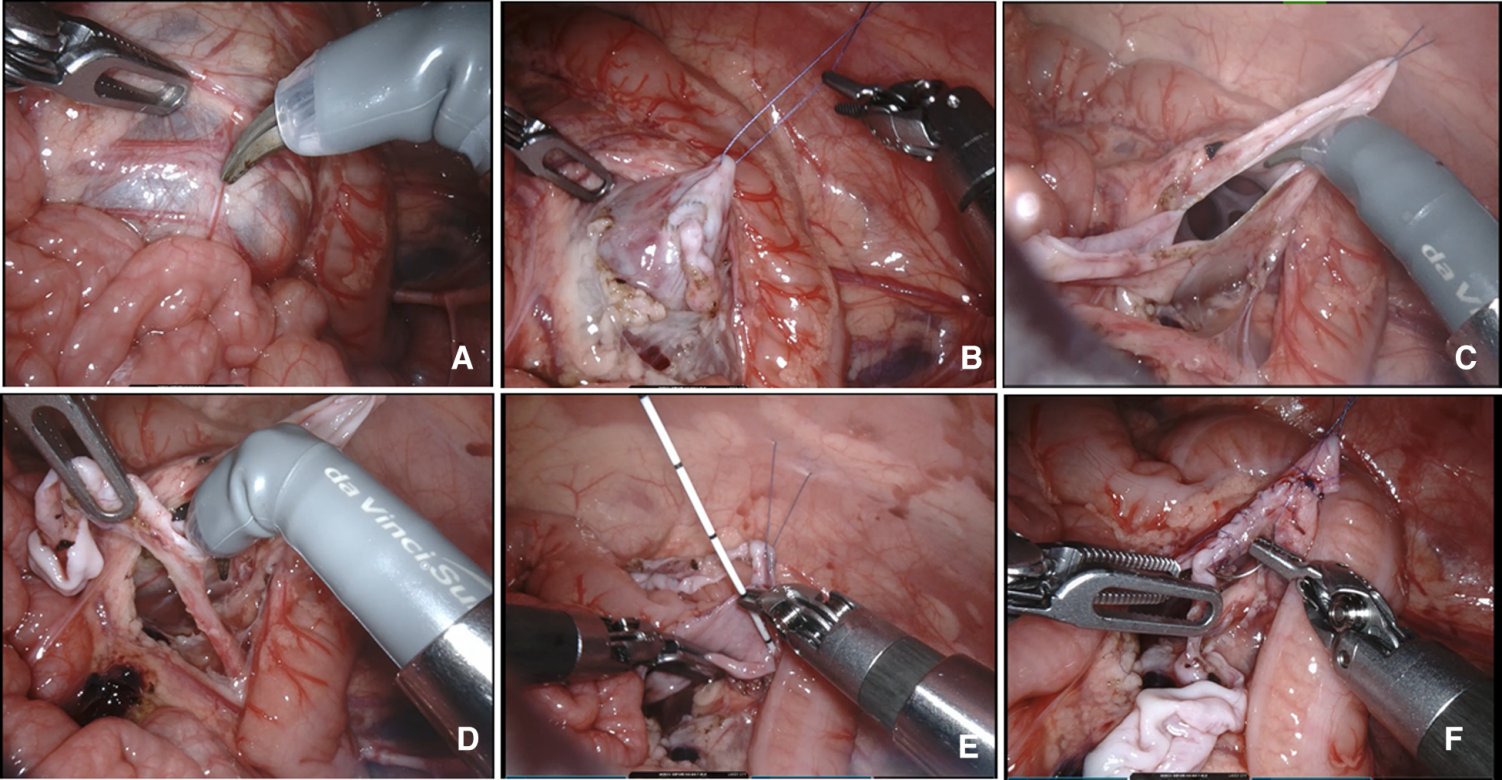
Surgical pyeloplasty procedures in the robotic groups. (**A**) The renal pelvis was identified by its dilation underneath the peritoneum. (**B**) A transabdominal hitch stitch was used to provide traction and facilitate exposure to the operative field. (**C**) Incision of the dilated renal pelvis. (**D**) Lateral spatulation of the ureter. (**E**) Anastomotic suture and insertion of the JJ stent. (**F**) Suturing and anastomosis of the anterior wall.

After the artificial pneumoperitoneum was established and the instruments had been placed, the following surgical steps in each group were almost identical. These OUPJ cases were exposed transmesenterically, and no cases were found to be obstructed by the lower-pole vessel (Figure 2). The stenosis at the ureteropelvic junction was relieved and an anastomosis was performed *via* uninterrupted sutures manner using Johnson 6-0 PDS sutures. The JJ tube was inserted antegrade. Finally, the peritoneal incision was sutured. The major procedures of robot-assisted pyeloplasty are shown in Figure 2. All robotic and laparoscopic surgeries were performed by the same surgeon. No case was converted to an open procedure and no additional trocar was required in any of them.

### Patients' management after surgery

Once the urine no longer showed signs of bleeding, the urinary catheter was removed, generally within 3 days. If there was no anastomotic leakage and there were no signs of infection, a liquid diet was initiated after surgery, generally no more than 3 days. The parents' satisfaction with the cosmetic outcomes was evaluated by a visual analog scale 3 months after surgery. A diuretic nephrogram, renal static imaging, and magnetic resonance imaging were planned for 6 or 12 months after surgery, and an ultrasound of the urinary system was planned for 1, 3, 6, 9, and 12 months after surgery. The JJ tube was removed 1 month after surgery. Overall, 11, 1, 5, and 2 patients were lost to follow-up or did not return to the hospital on time for reexamination in LMPY, LSPY, RMPY, and RSPY, respectively.

### Data collection

Patients' characteristics—including age, gender, weight, and height—were recorded. Preoperative data—including SFU grade, differential renal function (DRF), anterior and posterior diameter of the renal pelvis and ureter (APDRPU)—were recorded. Operative data—including surgical site, operative time (skin to skin), trocar insertion time, urethroplasty time, JJ tube insertion time, ratio of urethroplasty time and total operative time, blood loss, transfusions, and volumes—were recorded. Follow-up data—including postoperative SFU, change of pre-postoperative SFU, postoperative DRF, change of pre-postoperative DRF, postoperative APDRPU, change of pre-postoperative APDRPU, duration of JJ tube and gastric tube use, duration of fasting, length of hospital stay, complications (urine leakage, infection, anastomotic stenosis), parents' satisfaction scores, and outcomes (3 months after surgery)—were collected and recorded. The data regarding bilateral OUPJ have been divided and averaged into unilateral.

### Statistical analysis

Values are presented as median (range) when continuous variables do not conform to the normal distribution; otherwise, mean ± standard deviation is employed. Categorical variables are presented as numbers. Analyses were performed by SPSS (SPSS, statistics, version 21.0, IBM Corp., New York City, NY, USA), categorical variables and nonnormal distribution continuous variables were evaluated by nonparametric analysis (chi-square or Kruskal–Wallis H test) and normal distribution continuous variables were evaluated by test two-way ANOVA. A *P*-value < 0.05 was considered statistically significant.

## Results

The number of patients in groups LMPY, LSPY, RMPY, and RSPY were 31, 5, 28, and 10, respectively. The patients' demographic characteristics—including age, gender, height, and weight—did not show significant differences (Table 1). Most of the patients were diagnosed with unilateral OUPJ and received a unilateral pyeloplasty. One patient in each of the following three groups (LMPY, RMPY, and RSPY) was diagnosed with bilateral OUPJ and received a bilateral pyeloplasty. However, there was no statistical difference (*P* = 0.20; Table 1). Before surgery, the patients' SFU grades stood at III or higher, and the number of various SFU states in each group showed no difference (*P *= 0.90; Table 1). The APDRPU of the four groups before the operation was not statistically different, even though it was lower in the RMPY group than in the other three groups (*P *= 0.055; Table 1). In addition, the preoperative DRF in each group showed no statistical difference (*P *= 0.29; Table 1).

**Table 1:** Patients’ characteristics and preoperative data of UPJO.

	LMPY	LSPY	RMPY	RSPY	*P*
Age at surgery					
Median (range, year)	0.90 (0.11, 12.0)	3.0 (0.53, 9.0)	1.50 (0.11, 14.0)	2.50 (0.17, 11.0)	0.44
Gender					0.51
Male	25	3	23	7	0.67
Female	6	2	5	3	
Height at surgery, Median (range, cm)	71.0 (52.0, 157.0)	100.0 (66.0, 130.0)	82.5 (55.0, 176.0)	92.0 (56.0, 148.0)	0.35
Weight at surgery, Median (range, kg)	9.60 (4.60, 45.0)	15.0 (8.0, 25.0)	11.50 (5.0, 75.0)	13.90 (5.10, 43.0)	0.71
Surgical side					
Left	21	3	19	5	0.20
Right	9	2	8	4	
Bilateral	1	0	1	1	
Preoperative SFU					0.90
III	11	2	13	4	
IV	20	3	15	6	
Preoperative APDRPU	71.88 ± 17.69	62.20 ± 19.95	55.36 ± 17.62	67.05 ± 30.48	0.055
Preoperative DRF	36.97 ± 7.84	31.77 ± 9.05	38.29 ± 7.57	34.11 ± 10.90	0.29
Following time, median (range, month)	12 (3, 12)	12 (6, 12)	9 (3, 12)	9 (3, 12)	0.072

LMPY, laparoscopic multiport pyeloplasty; LSPY, laparoscopic single-port pyeloplasty; RMPY, robot-assisted multiport pyeloplasty; RSPY, robot-assisted single-port-plus-one pyeloplasty; SFU grade, Society of Fetal Urology grade; APDRPU, anterior and posterior diameters of the renal pelvis and ureter; DRF, differential renal function.

There was a significant difference in the operative time and trocar insertion time between the four groups. The operative time of the LMPY group was less than that of the other three groups, and the whole operative times of the single-port groups (LSPY and RSPY) were significantly longer than those of the multiport groups (LMPY and RMPY) (*P *< 0.001; Table 2). There was a statistical difference in the ureteroplastic times. The ureteroplastic time of the LSPY group was significantly shorter than that of the LMPY group (*P *= 0.0076; Table 2). Although the final comparison between the RSPY and RMPY groups did not show such a difference, the ratio of ureteroplastic formation time and whole operative time (UT/WT) showed a significant difference between the robotic groups (RMPY and RSPY) and the laparoscopic groups (LMPY and LSPY). There was no significant difference in the JJ tube insertion time and volume of blood loss (Table 2), and no patients required a blood transfusion.

**Table 2:** Patients’ intraoperative data.

	LMPY	LSPY	RMPY	RSPY	*P*
Trocar insertion time	7.84 ± 1.73	12.80 ± 1.79	17.10 ± 3.02	23.00 ± 2.40	<0.001
Whole operative time (skin to skin)	119.58 ± 46.61	212.0 ± 98.27	164.93 ± 29.72	169.80 ± 18.88	<0.001
Ureteroplastic formation time	78.97 ± 37.38	149.40 ± 86.38	88.75 ± 39.17	89.50 ± 18.87	0.0076
Ratio of UT/WT	0.64 ± 0.075	0.67 ± 0.11	0.53 ± 0.20	0.53 ± 0.09	0.0075
JJ tube insertion time	3.45 ± 2.31	3.40 ± 1.14	3.0 ± 1.05	2.90 ± 0.99	0.70
Volume of blood loss	16.35 ± 25.50	8.60 ± 4.16	6.28 ± 3.18	6.10 ± 2.28	0.11

LMPY, laparoscopic multiport pyeloplasty; LSPY, laparoscopic single-port pyeloplasty; pyeloplasty; UT, ureteroplastic formation time; WT, whole operative time (skin to skin).

**Figure 3:**
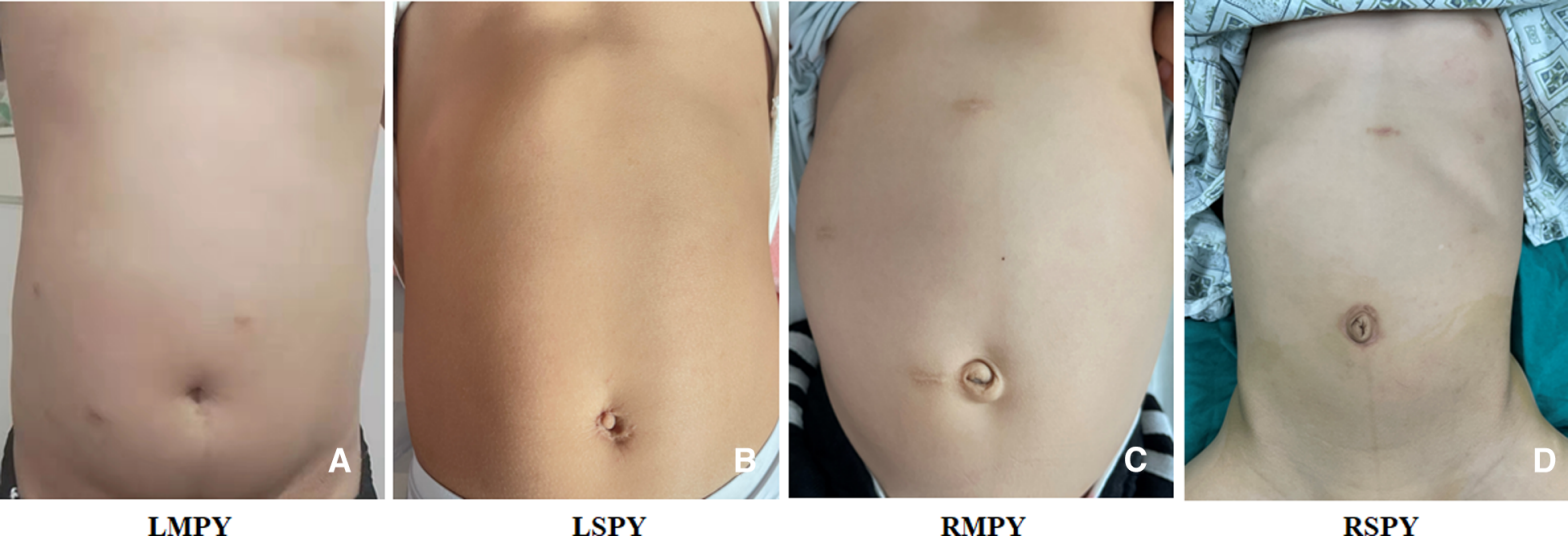
The cosmetic outcome of the surgical incision in each group. (**A**) LMPY, laparoscopic multiport pyeloplasty. (**B**) LSPY, laparoscopic single-port pyeloplasty. (**C**) RMPY, robot-assisted multiport pyeloplasty. (**D**) RSPY, robot-assisted single-port-plus-one pyeloplasty.

The fasting time (*P *= 0.89; Table 3) and ureteral catheter retainment time (*P *= 0.34; Table 3) of the groups showed no significant difference. However, the hospitalization times of the robotic groups (RMPY and RSPY) were shorter than those of the other groups (*P *< 0.001; Table 3). At postoperative follow-up, there was no significant difference in the SFU grade (*P *= 0.17; Table 3) or the pre-post change in the SFU grade (*P *= 0.23; Table 3). In addition, there was no statistical difference in the postoperative APDRPU and the pre-post APDRPU among these groups (Table 3). Complications—including anastomotic leakage and urinary tract infection—were not significantly different among the groups (Table 3), and anastomotic stenosis did not occur in any group. Finally, the parents' satisfaction scores regarding the cosmetic outcomes of surgery were significantly higher in the single-port groups (LSPY and RSPY) than those in the multiport groups (LMPY and RMPY) (*P *< 0.001; Table 3). The cosmetic outcomes are shown in Figure 3.

**Table 3:** Patients’ postoperative follow-up data.

	LMPY	LSPY	RMPY	RSPY	*P*
Fasting time	2.13 ± 0.76	2.20 ± 0.84	2.0 ± 0.77	2.0 ± 0.82	0.89
Ureteral catheter retainment time	3.13 ± 2.48	2.50 ± 1.58	2.43 ± 2.63	1.70 ± 0.82	0.34
Hospitalization time	11.84 ± 4.40	9.60 ± 6.19	5.71 ± 2.42	5.80 ± 2.57	<0.001
Postoperative SFU grade					0.17
0	5	1	3	0	
I	5	2	8	1	
II	10	1	11	7	
III	0	0	1	0	
Pre-post-SFU grade					0.23
I	3	0	4	3	
II	10	2	11	5	
III	6	2	8	0	
IV	1	0	0	0	
Postoperative DRF	46.28 ± 2.63	45.81 ± 1.63	46.19 ± 3.01	45.0 ± 4.16	0.76
Pre-post change in DRF	9.33 ± 5.95	15.75 ± 8.54	7.28 ± 4.94	11.70 ± 9.40	0.071
Postoperative APDRPU	21.10 ± 8.40	19.25 ± 2.99	22.96 ± 10.79	23.0 ± 9.91	0.84
Pre-post change in APDRPU	39.80 ± 12.87	36.50 ± 22.34	34.91 ± 13.92	34.88 ± 19.52	0.75
Parents’ cosmetic satisfaction scores	8.58 ± 0.81	9.60 ± 0.55	8.39 ± 0.92	9.40 ± 0.52	<0.001
Complications (YES/NO)	4/27	0/5	3/25	1/9	1.00
Anastomotic leakage (YES/NO)	1/30	0/5	1/27	0/10	1.00
Urinary tract infection (YES/NO)	3/28	0/5	2/26	1/9	1.00

LMPY, laparoscopic multiport pyeloplasty; LSPY, laparoscopic single-port pyeloplasty; RMPY, robotic-assisted multiport pyeloplasty; RSPY, robotic-assisted single-port-plus-one pyeloplasty; SFU grade, Society of Fetal Urology grade; APDRPU, anterior and posterior diameter of the renal pelvis and ureter; DRF, differential renal function; pre-post-SFU change, the change in SFU grade before and after surgery.

## Discussion

The first reported robot-assisted urologic surgery in a child was performed by Dr. Craig Peters of Boston Children's Hospital in 2002 (8). After 20 years of development, more and more laparoscopic surgeries were completed with robotic assistance. According to Sameer (9), more than 75% of pyeloplasties and reimplants are now performed robotically. The concept of MIS is getting more and more attention, especially in pediatric procedures, and the use of a single port offers a new option for pyeloplasty. However, pure single-port surgery in a toddler or an infant less than 1 year of age is more difficult. There are studies reporting single-port and robotic procedures in pediatric pyeloplasty, but no one has yet compared the results of single-port and robotic surgery vs. robotic and laparoscopic surgery. In the present study, we employed a new method of trocar location and compared the perioperative and follow-up effects of different approaches to the placement of trocars in laparoscopic and robotic procedures.

In our study, the whole time of operation in the RSPY group was significantly less than that in the LSPY group but was not significantly different from that in the RMPY group. However, the use of a single port clearly decreased the operative time as compared with the use of multiple ports, as in Kang's report; this difference was reduced in our last 15 cases (10). In our experience, the use of a single port did not simplify surgical procedures. In Kang's study, the patients were mainly between 4 and 10 years of age; this may have meant that there was much more working space, which could have accelerated the operative process. Even the trocar placement and ureteroplastic formation time in the robotic groups were obviously longer than those in the laparoscopic groups. That is, the ratio of ureteroplastic formation time and the whole operative time was significantly smaller in the robotic groups. The ratio of UT/WT is a good indicator of the advantages of robotic surgery and the advantages of robotic instruments in small-space operations. Furthermore, the difference between the robotic and laparoscopic groups may be explained by the learning curve of the operator. The suggested case of robotic-assisted pyeloplasty for the operator to achieve the median operative time is about 40 according to the previous reports (11, 12). In addition, the median time of robotic-assisted single-port surgery may be longer in an infant under 1 year of age owing to the smaller abdominal working space. This conclusion is consistent with Andolfi's report (13).

Pyeloplasty was first described by Anderson-Hynes and has become the gold standard intervention for OUPJ (14, 15). It can be performed by open, laparoscopic, or robot-assisted approaches. In addition to early recovery, shorter hospital stays, and better cosmetic result, the surgical success rate associated with robot-assisted pyeloplasty is its most important advantage. The resolution rate in both the robotic and laparoscopic approaches is greater than 90%, as reported earlier (13, 16–19). In our center, the overall resolution rate in the four surgical groups has reached 98.2% (in terms of the postoperative SFU grade); only one patient. In RMPY, it remained at SFU grade III following surgery. In addition, in all groups, there was no significant difference in the improvement of APDRPU and DRF. This result was similar in both RSPY and RMPY, which suggests the potential benefit of robotic single-port pyeloplasty in pediatric urology.

The advantages of robotic surgery include improved dexterity and precision, three-dimensional high-definition vision, and the filtration of tremors (20). In this study, the fasting and ureteral catheter retainment times were shorter in the robotic groups than in the laparoscopic groups. Also, there was a significant difference in hospitalization time (including the days before and after surgery) among these groups. The rates of perioperative and postoperative complications among the groups showed no significant differences.

Whether a 25- to 30-mm single incision and a single port are a benefit in children has been controversial, especially with regard to infants less than a year old. In our study of laparoscopic single-port pyeloplasty, we tried to improve the port placement protocol by using one additional trocar. However, we must emphasize that the robotic arm must be pushed through the port with special care because the child's abdomen is particularly pliable. Our technical tips regarding robotic surgery in infants include the following. First, the surgeon should have completed 30 or more robotic surgeries in older children before approaching another in an infant. Second, it is safer to insert the trocar under direct vision and manual control with the camera. Third, the placement of an orogastric and/or rectal tube to reduce bowel distention may help in exposing the working space. Finally, the abdominal cavity should be closed layer by layer, starting from the peritoneum to avoid the creation of an incisional hernia.

In conclusion, this study documents some of the comparative resolution rates and benefits of robotic and single-port pediatric pyeloplasty. However, its conclusions would be more convincing if a similar study were to be performed with larger sample size and longer follow-up period. Also, a more detailed study focusing on the patients' age may lead to more reliable conclusions.

## Conclusion

Our study shows that both robotic multiple-port and single-port-plus-one approaches are comparable to laparoscopic multiple-port and single-port approaches in terms of resolving OUPJ in children. The robotic approaches may offer some advantages in terms of hospitalization time and cosmetic outcomes. The accuracy of the robotic approach may be of benefit in urologic surgery that requires precise suturing, especially in pediatric patients.

## Data Availability

The original contributions presented in the study are included in the article/Supplementary Material, further inquiries can be directed to the corresponding author/s.
