# Corrigendum: Robot-assisted pyeloplasty and laparoscopic pyeloplasty in children: a comparison of single-port-plus-one and multiport surgery

**DOI:** 10.3389/fped.2025.1559673

**Published:** 2025-01-22

**Authors:** Jianglong Chen, Huihuang Xu, Shan Lin, Shaohua He, Kunbin Tang, Zhixiang Xiao, Di Xu

**Affiliations:** ^1^ Shengli Clinical Medical College, Fujian Medical University, Fuzhou, China; ^2^ Department of Pediatric Surgery, Fujian Provincial Hospital, Fuzhou, China

**Keywords:** pediatric, ureteropelvic junction obstruction, pyeloplasty, robotic, single-port

A corrigendum on Robot-assisted pyeloplasty and laparoscopic pyeloplasty in children: a comparison of single-port-plus-one and multiport surgery By Chen J, Xu H, Lin S, He S, Tang K, Xiao Z and Xu D (2022). Front. Pediatr. 10:957790. doi: 10.3389/fped.2022.957790

In the published article, there was an error in the order of affiliations 1 and 2. Instead of “1 Department of Pediatric Surgery, Fujian Provincial Hospital, Fuzhou, China, 2 Shengli Clinical Medical College, Fujian Medical University, Fuzhou, China”, it should be “1 Shengli Clinical Medical College, Fujian Medical University, Fuzhou, China, 2 Department of Pediatric Surgery, Fujian Provincial Hospital, Fuzhou, China”.

The authors apologize for this error and state that this does not change the scientific conclusions of the article in any way. The original article has been updated.

